# Primary testicular non-Hodgkin's lymphoma – a review article

**DOI:** 10.1590/S1516-31802007000500007

**Published:** 2007-09-02

**Authors:** Ashok Kumar Vaid, Sachin Gupta, Dinesh Chandra Doval, Vineet Talwar

**Keywords:** Lymphoma, Testis, Central nervous system, Injections, Orchidectomy, Genital neoplasms, male, Linfoma, Testículo, Sistema nervoso central, Injeções, Neoplasias da genitália masculina, Orquiectomia

## Abstract

Primary testicular non-Hodgkin's lymphoma was first described as a clinical entity in 1866. It is a rare disease and accounts for 1% of all non-Hodgkin's lymphoma, 2% of all extranodal lymphomas and 5% of all testicular neoplasms. It is the most common testicular tumor in males between sixty and eighty years of age. Testicular non-Hodgkin's lymphoma is unique in its high incidence of bilateral involvement (8-38%), and it is also the most common bilateral testicular tumor. Testicular non-Hodgkin's lymphoma has a predilection for spreading to non-contiguous extranodal sites, especially the central nervous system. Advanced-stage disease is usually managed with doxorubicin-based chemotherapy. For early-stage disease, opinion is divided regarding systemic chemotherapy following orchidectomy. The high incidence of spreading, especially to the central nervous system, leads to advocacy of the use of central nervous system prophylaxis with intrathecal chemotherapy. Prospective multicenter trials incorporating a large number of patients may lead to better guidelines for optimal management of this subtype of non-Hodgkin's lymphoma.

## RESUMO

O linfoma primário do testículo (LPT) foi descrito como uma entidade clínica pela primeira vez em 1866. É uma doença rara e corresponde a 1% de todos os linfomas não-Hodgkin, 2% de todos os linfomas extranodais e 5% de todos as neoplasias testiculares. É o tumor testicular mais comum em homens entre 60 e 80 anos de idade. LPT é único em sua elevada incidência de envolvimento bilateral (8-38%), sendo o tumor testicular bilateral mais comum. Tem uma predileção por disseminação para regiões extranodais não-contíguas, especialmente para o sistema nervoso central (SNC). Estágios avançados da doença são usualmente tratados com quimioterapia à base de doxorubicina. Para os estágios mais precoces, as opiniões são divergentes quanto à quimioterapia associada à orquiectomia. A alta prevalência de disseminação, especialmente para o SNC, sugere o uso de quimioterapia intratecal como profilaxia. Estudos prospectivos multicêntricos incluindo um grande número de pacientes poderiam resolver a questão com relação ao manejo deste subtipo de linfoma não-Hodgkin.

## INTRODUCTION

Although testicular non-Hodgkin's lymphoma (TNHL) is a rare entity and constitutes only 1-7% of all testicular neoplasms, it is the most common testicular tumor in males between sixty and eighty years of age. TNHL is unique in its high incidence of bilateral involvement (8-38%), and it is also the most common bilateral testicular tumor. TNHL has a predilection for spreading to non-contiguous extranodal sites, especially the central nervous system (CNS).^1–6^ The majority of reported TNHL cases present diffuse histology, and usually B-cell type.^1,3,4,7–9^ As it is a rare tumor, and population-based studies are few, its treatment is controversial, especially regarding its early stage.^10^ However, the use of systemic chemotherapy following orchidectomy is a standard treatment for advanced disease.^7,10,11^ The prognosis for TNHL is poor, particularly if disseminated disease is evident within one year after diagnosis.^5,12^ Also, CNS prophylaxis with intrathecal chemotherapy is advocated by many authors, in view of the fact that the majority of patients present relapse within the first two years following treatment, and such recurrence is especially in the CNS.^10,13–15^

## EPIDEMIOLOGY AND INCIDENCE

Primary TNHL was first described as a clinically entity in 1866.^1,3^ It is a rare disease and accounts for 1% of all non-Hodgkin's lymphoma (NHL) cases, 2% of all extranodal lymphomas and 5% of all testicular neoplasms.^16^ However, secondary testicular involvement is not uncommon in advanced NHL cases, either as part of the terminal disease or in autopsy findings, and up to 20% of patients dying due to disseminated NHL have been reported to have microscopic testicular invasion.^4,15,17^

## AFFECTED AGE GROUPS AND BILATERALITY

TNHL usually occurs in older men and is the most common testicular malignancy in men between sixty and eighty years of age. High incidence of bilateral testicular involvement, which is a unique feature of TNHL, was first reported by Abeshouse et al. in 1955.^1^ TNHL is now considered to be the most common bilateral tumor of the testes,^1–6^ with reported incidence of bilateral metachronous testicular involvement of 35% and bilateral synchronous testicular involvement of 3%.^6^ Other authors have reported the incidence of bilateral involvement of about 10-40%.^7,12,15^

## POSSIBLE MULTICENTER ORIGIN

The prognosis is generally poor, with progressive systemic lymphomatous involvement.^5,6,8,10,11,15^ The short disease-free interval and poor prognosis usually point towards a multicenter origin for systemic TNHL.^6,8,9,13^ The hypothesis that TNHL has a multicenter origin is further supported by the high incidence of bilateral involvement, since there is no direct lymphatic or venous connection between the right and left testes.^6^ Many authors have classified TNHL as a secondary manifestation of occult systemic lymphomatous malignancy. It has been proposed that considering TNHL to be secondary may lead to early extensive evaluation of the occult disease, as well as leading to the possibility of curing the disease through more intensive systemic therapy along with local radiotherapy or orchidectomy.^6^ At the same time, the fact that there are patients who have had localized disease and have been cured through orchidectomy alone favors the existence of TNHL as primary disease. Jackson et al. reviewed 17 TNHL patients with localized disease, who had mostly either been cured or presented long survival without evidence of dissemination.^9^ These findings support the notion that TNHL is a clinical entity originating primarily in the testis and is not just the initial manifestation of subsequent generalized lymphomatous disease.

## ETIOLOGICAL FACTORS AND DISSEMINATION PATTERNS

There are neither any well-documented etiological or predisposing factors nor any significant associations existing between histories of trauma, chronic orchitis or cryptorchidism and subsequent development of TNHL.^1,4,6,9^ TNHL has a predilection for dissemination to non-contiguous extranodal sites such as the CNS, Waldeyer's ring, skin and lungs.^1,4,10,12^ Martenson et al. reported a 10% incidence of CNS involvement in the initial failure and 20% in subsequent relapses.^18^ CNS relapses have been reported in 14% of stage I cases and 10.5% of stage II cases.^10^ In a series of 22 patients, the CNS or the contralateral testis were involved in all the patients who failed to respond to primary therapy and in 50% of those who relapsed from complete remission.^16^ Thus, lumbar puncture is warranted as one of the initial procedures at the time of diagnosis.^4,15^

## HISTOLOGICAL CLASSIFICATION

According to the Working Formulation of the United States National Cancer Institute, approximately 68% of TNHL cases are classified as intermediate grade, diffuse large B-cell subtype, followed by high-grade, diffuse small non-cleaved subtype in about 30% of the patients.^1,12^ There is no prognostic advantage for any pathological subtype.^12^ Immunohistochemistry (IHC) studies confirm the majority of TNHL cases to be of B-cell origin, with lesser occurrence of T-cell lymphoma.^1,13^ Histopathological differentiation of TNHL from seminoma is usually a challenge.^6^ Other conditions that might resemble TNHL are embryonal cell carcinoma, granulomatous orchitis, pseudolymphoma, plasmacytoma and rhabdomyosarcoma.^1,3,6^ Serum lactate dehydrogenase (LDH) levels have been correlated with tumor aggressiveness,^13^ whereas other tumor markers such as serum beta human chorionic gonadotropin (HCG) and serum alpha-fetoprotein (AFP) are rarely elevated in TNHL cases.^13^ Al-Abbadi et al. studied 18 patients and classified their disease as primary testicular lymphoma with germinal center B-cell-like and non-germinal center B-cell-like by means of the IHC expression of CD10, Bcl-6 and MUM1. They found that 89% of the primary testicular lymphoma of the diffuse large B-cell type belonged to the non-germinal center B-cell-like subgroup and all exhibited high proliferative activity. The germinal center B-cell-like type of primary testicular lymphoma was uncommon and was seen mostly in HIV-positive patients.^19^

## TREATMENT

### Early-stage disease

Although there is enough data on TNHL and its management, there are very few population-based studies, and thus the treatment has not been standardized.^10^ For stages IE and IIE, there is universal agreement on orchidectomy as the initial treatment. If treated with orchidectomy alone, the majority of these patients relapse within the first two years at various extranodal sites, and hence the use of adjuvant systemic chemotherapy, radiotherapy and also prophylactic intrathecal chemotherapy has been emphasized.^10,20^ In a series of 16 patients, the median survival exceeded 57 months for those with stage IE disease, whereas it was six months for patients with advanced disease. No patient with disease that had spread to or beyond the para-aortic lymph nodes survived beyond 19 months.^12^ According to the same study, for stage IIE, radical irradiation offered the potential for cure, for all the pathological subtypes. In a similar study, Connors et al. reported that the four-year actuarial survival was 93% in a group of 15 patients with stage IE and IIE disease who were treated with orchidectomy and doxorubicin-based chemotherapy, with prophylactic radiotherapy for the uninvolved contralateral testis.^14^ In contrast to the data from Connors et al.^14^ and Tepperman et al.,^12^ another report on 24 patients showed that 10 out of 12 patients who underwent orchidectomy alone died of disseminated disease, and 50% died within the first six months following orchidectomy. The patients with stage IE and IIE disease who were treated with orchidectomy and irradiation of regional lymph nodes also presented an association with relapse.^7^

Stage IE is unique in that it is potentially curable in small subpopulations by means of orchidectomy alone. On the other hand, in other groups, late relapse and distant spreading that was non-responsive to retreatment with irradiation or chemotherapy has been evident. However, routine chemotherapy may not be recommendable in stage IE disease.^12^ According to a study on 34 patients registered with the British National Lymphoma Investigation (BNLI), adjuvant chemotherapy using the cyclophosphamide, doxorubicin, vincristine and prednisolone (CHOP) protocol was insufficient to prevent CNS relapse.^15^ In a Danish population-based NHL registry report, 24 out of 39 patients had localized disease. Early-stage patients who were treated with orchidectomy and doxorubicin-based chemotherapy had a relapse rate of 15.4%, while for those not given adjuvant chemotherapy, the relapse rate was 63.6%. The median relapse-free survival was 28 and 14 months and the overall two and five-year survival rates were 43% and 17%, respectively, for these two groups of patients. The overall complete remission rate for stages IE and IIE was 96%, in contrast to 7% for stage IV disease.^13^

Failure-free survival of 16% at 153 months was reported for a group of 22 patients at the MD Anderson Cancer Center.^16^ Either the CNS or the contralateral testis was involved in all the patients who failed to respond to primary therapy and in 50% of those who relapsed from complete remission. In that study, relapsed patients had received neither prophylactic intrathecal chemotherapy nor irradiation for the contralateral testis.^16^ In a similar review of 29 patients, all 16 with limited disease received doxorubicin-based chemotherapy following orchidectomy.^11^ Prophylactic radiotherapy for regional lymph nodes was given to 85% of the patients with limited disease. After a median follow-up of seven years, only one-third of patients with early-stage and slightly more than 10% of patients with advanced disease were alive.^11^

In a large series of 84 patients, 50% had stage IE disease.^10^ The treatment groups were divided into surgery only, surgery + radiotherapy, surgery + chemotherapy and surgery + radiotherapy + chemotherapy. None of the patients received CNS prophylaxis. At the end of the initial treatment, 61 (72.6%) achieved complete remission. Out of these, 32 patients relapsed and the most frequent site of recurrence was the CNS. Also, 50% of the stage IE patients relapsed. According to that study, there was no significant improvement in overall survival, disease-specific survival (DSS) or disease-free survival (DFS) in cases of stage IE disease when treated with orchidectomy alone, or when combined with chemotherapy or radiation. The median overall survival and DFS in that study were 32 months and 36 months respectively.^10^

Thus, treatment of early stage TNHL is controversial. Irradiation therapy alone is also not considered effective in view of the high rate of dissemination.^1,10^ Similarly, treatment with adjuvant doxorubicin has a high relapse rate despite achieving complete remission for the majority of patients.^1^

### Advanced-stage disease

For stage IIIE and IVE disease, the treatment of choice is systemic chemotherapy, with irradiation reserved for symptomatic and bulky localized deposits.^12^ Although the majority of patients achieve complete remission, most of them relapse, with a median survival of three to five months.^10^ In view of the high risk of CNS relapse, even in patients who achieve complete remission with primary therapy, CNS prophylaxis should be considered in all advanced-stage patients.^13^ Similarly, prophylactic irradiation for the contralateral testis is recommended, since the relapse rate in the contralateral testis is up to 50%.^16^ Nevertheless, contrary to this approach, some authors argue against prophylactic radiotherapy for the contralateral testis.^11^

## CONCLUSION

Thus, to summarize, TNHL is a rare tumor of the testes, with an incidence of only 1 to 7%. Nonetheless, it is the most common testicular tumor in older males. TNHL is unique in its propensity for bilateral involvement and is the most common bilateral testicular tumor. The prognosis is generally poor, since disseminated disease is usually evident within the first two years following the diagnosis. Advanced-stage disease is usually managed with doxorubicin-based chemotherapy. For early-stage disease, opinion is divided regarding systemic chemotherapy following orchidectomy. The high incidence of spreading, especially to the CNS, leads to advocacy of the use of CNS prophylaxis with intrathecal chemotherapy. Prospective multicenter trials incorporating a large number of patients would lead to better treatment options for this subtype.
